# Differential Profile of Hemostasis in Dengue Fever Before and After COVID-19

**DOI:** 10.3390/v17111431

**Published:** 2025-10-28

**Authors:** Alanna Calheiros Santos, Julya Farias Carneiro, Anna Paula de Souza e Silva Sales, Mariana Gandini, Flávia Barreto dos Santos, Paulo Vieira Damasco, Luis José de Souza, Elzinandes Leal de Azeredo, Luzia Maria de-Oliveira-Pinto

**Affiliations:** 1 Laboratório das Interações Vírus-Hospedeiros, Instituto Oswaldo Cruz, Fundação Oswaldo Cruz, Rio de Janeiro 21040-360, Brazil; julyafc28@gmail.com (J.F.C.); flaviab@ioc.fiocruz.br (F.B.d.S.); elzinandes@ioc.fiocruz.br (E.L.d.A.); 2 Centro de Doenças Imuno-Infecciosas, Hospital dos Plantadores de Cana, Campos dos Goytacazes, Rio de Janeiro 28025-496, Brazil; ap.sss@hotmail.com (A.P.d.S.eS.S.); luizjosedes@gmail.com (L.J.d.S.); 3 Laboratório de Microbiologia Celular, Instituto Oswaldo Cruz, Fundação Oswaldo Cruz, Rio de Janeiro 21040-360, Brazil; gandini@ioc.fiocruz.br; 4 Rede Casa Hospital Rio Laranjeiras e Rio Botafogo, Rio de Janeiro 22240-000, Brazil; paulovieiradamasco@gmail.com; 5 Departamento de Doenças Infecciosas e Parasitárias, Escola de Medicina e Cirurgia, Universidade Federal do Estado do Rio de Janeiro, Rio de Janeiro 20270-004, Brazil

**Keywords:** dengue, COVID-19, coagulopathy

## Abstract

Dengue and COVID-19 are viral diseases characterized by coagulopathies, with Dengue associated with fibrinolysis and COVID-19 with prothrombotic events. Furthermore, cross-reactivity between anti-SARS-CoV-2 and anti-DENV antibodies may confer protective immunity or exacerbate disease severity. Our investigation explored the impact of prior COVID-19 exposure on the immunopathogenesis of Dengue, focusing on hemostatic parameters. We quantified nitrites, procoagulant, anticoagulant, and fibrinolytic mediators in the plasma of Dengue patients before and after the COVID-19 pandemic. We also evaluated the influence of plasma from dengue patients on platelet activation in vitro using platelets from healthy donors exposed to DENV-2. Hemorrhagic manifestations were more frequent in pre-COVID-19 Dengue, while nitrite levels were elevated in post-COVID-19 Dengue patients, particularly among those without hemorrhagic signs. Among procoagulant factors, tissue factor (TF) levels were increased in post-COVID-19 Dengue, whereas Factor XIII was higher in pre-COVID-19 Dengue. In contrast, antithrombin (an anticoagulant) and plasminogen (a profibrinolytic factor) were more elevated in pre-COVID-19 Dengue than in post-COVID-19 cases. In vitro, DENV-2-infected platelets exposed to plasma of Dengue patients before and after COVID-19 showed decreased nitrite production in relation to DENV-2 alone. These findings suggest that prior COVID-19 exposure may influence hemostatic responses in Dengue, potentially modulating disease pathophysiology and opening new avenues for research and therapeutic strategies.

## 1. Introduction

During the COVID-19 pandemic, there was strong support for the high risk of dengue virus (DENV) and SARS-CoV-2 co-infections [1], especially in over 100 countries in tropical and subtropical areas where dengue is endemic, such as Southeast Asia, the Americas, and the Western Pacific region [2,3]. The risk of co-infections was expected to have an even more disastrous impact, given that these endemic regions faced other critical public health challenges, such as difficulty in accessing vaccination, socioeconomic inequalities, and the spread of misinformation, among others [4]. Brazil has the highest dengue incidence rates in South America [5], so this risk is a genuine concern for governments and health professionals in the country [6]. Brazil was brutally affected by the pandemic [7] that occurred just before the seasonal peak of Dengue, causing a simultaneous outbreak of two viruses in the first few weeks of 2020. However, a decrease in Dengue cases was also observed in other states [8]. Other dengue-endemic regions in Asia and the Americas have also seen a decline in dengue cases during the pandemic [9,10]. There are many theories to explain changes in Dengue transmission patterns during the pandemic, including mobility restrictions [8]. Furthermore, as the clinical features of COVID-19 and Dengue can be very similar, clinical misdiagnosis between COVID-19 and Dengue has occurred and been substantiated by false-positive results of rapid serological tests, particularly in Dengue endemic regions [11,12]. A study by Lokiada et al. evaluated a possible DENV co-infection in 42 patients with COVID-19. No cases of co-infection were identified, but an indication of recent DENV infection was seen by the presence of anti-DENV IgM and/or anti-DENV IgG levels in seven patients [13]. Our group in 2020 also investigated potential co-infections in 57 patients with COVID-19. None had viral load detected by RT-qPCR or viremia by DENV NS1 antigen. Still, five of them (8.8%) had anti-DENV IgM antibodies [14], corroborating the findings of Lokiada et al. in Indonesia. In this same study, we also saw that among Dengue patients recruited before COVID-19, in one of them (8.3%), anti-SARS-CoV-2 Spike 1 IgA was detected, and in none of them was anti-SARS-CoV-2 Spike 1 IgG detected, indicating that the commercial serological kits for COVID-19 used presented high specificity and sensitivity [14]. On the other hand, a study by Cheng et al. demonstrated that serum from COVID-19 patients showed neutralizing capacity against DENV infection in vitro and that this activity was blocked in the presence of the receptor-binding domain (RBD) of the Spike 1 (S1) protein, but not of the SARS-CoV-2 nucleocapsid protein, suggesting cross-reactivity of S1-RBD antibodies that may be involved in the inhibition of DENV infection in COVID-19 patients [15]. These findings are significant in understanding the immune response to co-infections.

The impact of cross-reactivity of anti-SARS-CoV-2 and anti-DENV antibodies on the severity of both diseases is still controversial. It is unclear whether antibodies induced by antigenic cross-reactivity between DENV and SARS-CoV-2 can provide overlapping protective immunity between these two diseases or aggravate the disease through the Antibody-Dependent Enhancement (ADE) phenomenon [16].

Dengue and COVID-19 exhibit similar clinical features and laboratory findings, particularly concerning coagulation dysregulation and inflammatory processes. Both viruses can lead to severe clinical complications [17]. Dengue fever disrupts hemostasis by increasing the activity of fibrinolysis, which hampers both primary and secondary hemostasis [18]. In contrast, COVID-19 is associated with an increase in prothrombotic events. Autopsies of patients with pneumonia have revealed the presence of macrovascular and microvascular thrombi in the lungs and other organs. Additionally, plasma concentrations of tissue plasminogen activator (tPA) and plasminogen activator inhibitor 1 (PAI-1) are significantly elevated in patients with COVID-19. Under normal physiological conditions, fibrinolytic agents such as tPA and urokinase plasminogen activator (uPA) work to prevent fibrin accumulation. However, in COVID-19, the elevated levels of PAI-1 and thrombin-activatable fibrinolysis inhibitors overshadow the effects of tPA and uPA, leading to increased fibrinolysis. The extremely high levels of D-dimers seen in COVID-19 patients are indicative of this heightened fibrinolytic activity [19]. Nitric oxide (NO) has been investigated as a potential therapeutic agent for treating thrombosis in COVID-19 due to its capacity to reduce platelet activation and adhesion [20]. In the case of dengue fever, an increase in vascular nitric oxide is linked to greater vascular permeability and impaired homeostasis, serving as a predictor for dengue hemorrhagic fever [21].

The process of blood coagulation begins with the exposure of tissue factors (TF) to the cells surrounding blood vessels, triggering the activation of the extrinsic pathway. In pathological conditions, endothelial cells express TF, mainly under inflammatory stimuli such as TNF-α and IL-1β, which contributes to the activation of coagulation and worsening of inflammatory processes [22]. This mechanism leads to the conversion of factor X into its active form, which converts prothrombin into thrombin, resulting in the formation of fibrin, a crucial element for preventing bleeding. The extrinsic pathway is stimulated by lesions that expose TF to the blood, allowing for its interaction with factor VII and the subsequent activation of factor X, initiating the activation of the coagulation cascade. On the other hand, the intrinsic pathway is activated when damage occurs to the vascular endothelium, allowing blood components to meet collagen or negatively charged surfaces. This contact triggers the activation of factor XII, which initiates a sequential cascade of activation of factors XI and IX. The common pathway represents the point of convergence between the intrinsic and extrinsic pathways, where activated factor X transforms prothrombin (factor II) into thrombin, which converts fibrinogen (factor I) into fibrin. Fibrin, the product of this complex process, forms a structural network stabilizing the clot, ensuring its hemostatic function and preventing excessive bleeding [23].

Our study aims to understand how COVID-19 affects the immunopathogenesis of dengue, particularly the frequency of hemorrhagic manifestations and changes in the mediators of the coagulation cascade in the post-pandemic period. To achieve this, we will quantify NO, procoagulant, anticoagulant, and fibrinolytic mediators in plasma samples from dengue patients before and after COVID-19. We will also evaluate the impact of plasma from dengue patients before and after exposure to COVID-19 and DENV-2 on platelet activation, specifically regarding CD62P expression and NO production.

## 2. Materials and Methods

### 2.1. Study Participants

This study consisted of two methodological components: (1) a retrospective cohort study, conducted with samples from patients with confirmed dengue diagnosis, collected in two distinct time periods: from 2016 to 2018 (pre-COVID-19 pandemic) and in 2023 (post-pandemic), using stored plasma samples and clinical data; and (2) a prospective experimental arm, in which platelets were obtained from healthy volunteer donors to evaluate in vitro platelet activation following exposure to DENV-2 and patient plasmas.

Retrospective cohort study with 85 participants, whose inclusion criteria were patients with a clinical diagnosis of dengue confirmed by laboratory tests for NS1 DENV, RT-qPCR DENV, and/or positive anti-DENV IgM. The study used blood plasma or serum samples from patients stored in the Virus-Host Interactions Laboratory at −80 °C, collected in dengue outbreaks prior to (2018) and after (2023) the COVID-19 pandemic. Patients were recruited from the Reference Center for Immunoinfectious Diseases in Campos dos Goytacazes and Rede-Casa hospitals in Rio de Janeiro. Dengue patients were categorized into two groups: (i) Dengue patients not exposed to SARS-CoV-2 (Dengue before COVID-19), composed of 32 patients diagnosed with dengue with no history of exposure to SARS-CoV-2, whose samples were collected in the 2018 epidemic (serotypes DENV-1, DENV-2 and DENV-4); and (ii) Dengue patients exposed to SARS-CoV-2 by natural infection and/or vaccination (post-COVID-19 Dengue), formed by 36 patients whose samples were collected in 2023. Reference values for hematological parameters were derived from a group of healthy blood donors (*n* = 17) included in this study. We acknowledge that these values are exploratory and do not substitute standard clinical laboratory reference ranges derived from anti-body-negative control populations. These donors presented no clinical signs or symptoms of infection or inflammation for 3 months or at the time of collection that occurred in 2024, and all presented positive serology for anti-SARS-CoV-2, either by natural infection/or vaccination. The use of healthy donors as a reference group for hematological comparisons represents a methodological limitation, as these values may not align with standard reference intervals provided by clinical laboratories. This limitation is inherent to the retrospective design and should be considered when interpreting comparisons.

Both the retrospective and prospective components of the study, which adhered to the highest ethical standards, were approved by the Sistema Plataforma Brasil’s Research Ethics Committee (CEP) under the numbers CAAE 57221416.0.1001. 5248.version 6 (12 May 2020). This approval underscores our commitment to conducting research that respects and protects the rights and welfare of our participants. All participants, including patients and healthy donors, provided written informed consent.

### 2.2. Detection of Molecular and Serological Markers for Laboratory Confirmation of Dengue

Viral RNA was extracted using the Biogen viral DNA/RNA extraction kit (Cat. no. K204, Bioclin) from plasma or serum samples, following the manufacturer’s protocol. Once extracted, RNA was stored at −70 °C until analysis. The RT-qPCR method performed using the GoTaq^®^ Probe 1-Step RT-qPCR System kit (Cat. no. A612X, Promega, Madison, WI, USA) was specific for the four DENV serotypes (1–4), CHIKV, and ZIKV [24]. Reactions were carried out on the Applied Biosystems 7500 real-time PCR system. The samples considered positive presented cycle threshold (Ct) values lower than 38, providing reliable results.

For serological diagnosis, the DENV anti-Dengue Virus IgM ELISA (Cat. no. EI 266a-9601-1M, EUROIMMUN, Lubeck, Germany), anti-Dengue Virus IgG ELISA (Cat. no. EI 266b-9601G, EUROIMMUN) and NS1 protein Platelia Dengue NS1 Ag (Cat. no. 72830, BioRad, Platelia, Hercules, CA, USA) kits were used. It is important to note that individuals with any positive result for CHIKV or ZIKV were rigorously excluded from the study.

### 2.3. Serological Markers of COVID-19

The detection of specific IgA and IgG antibodies against SARS-CoV-2 in the participants’ samples was performed by ELISA, using the Anti-SARS-CoV-2 IgA (Cat. no. EI 2606-9601-2) and Anti-SARS-CoV-2 IgG (Cat. no. EI 2606-9601-1) kits, both from EUROIMMUN. All procedures followed the manufacturer’s instructions.

### 2.4. Detection of Nitric Oxide (NO) by Griess Reaction

The quantification of nitric oxide (NO) was performed indirectly by detecting nitrite (NO_2_^−^) using the Griess reagent kit (Cat. no. G2930, Promega), according to the manufacturer’s instructions.

### 2.5. Enzyme-Linked Immunosorbent Assay for Quantification of Soluble Mediators TF and TFPI

According to the manufacturer’s instructions, the R&D Systems DuoSet ELISA kits, specific for Human Tissue Factor/Coagulation Factor III (Cat. no. DY2339) and TFPI (Cat. no. DY2974) were used.

### 2.6. Quantification of Fibrinolysis and Coagulation Regulatory Proteins by Multiplex Assay

The LEGENDplex™ Human Fibrinolysis Panel (5-plex) (Cat. no. 740761, BioLegend, San Diego, CA, USA) was used to simultaneously analyze five human proteins involved in coagulation and fibrinolysis: Fibrinogen, Antithrombin, Plasminogen, Prothrombin, and Factor XIII. This panel uses coded fluorescent beads, allowing for the identification and quantification of analytes based on fluorescence intensity. Samples were analyzed on a Beckman Coulter CytoFLEX S flow cytometer, following the manufacturer’s instructions.

### 2.7. Production of Viral Stock

The BR/RJ0337/2008 strain, belonging to DENV-2 Lineage II, was isolated in 2008 from a newborn with high titers of anti-DENV antibodies and clinical symptoms of mild dengue by the Flavivirus Laboratory of the Oswaldo Cruz Institute (IOC) of Fiocruz. Strain BR/RJ0337/2008 was inoculated into Aedes albopictus clone C6/36 cells to prepare the viral stock. The viral titer was determined by the Reed and Muench method [25], resulting in 10^9^ TCID50/mL after seven passages. The serotype was confirmed by indirect immunofluorescence, using a monoclonal antibody specific to DENV-2, as described by Gubler and collaborators [26].

### 2.8. Platelet Purification

Peripheral blood samples were meticulously obtained from healthy donors (*n* = 3) by venipuncture using 8.5 mL Vacutainer^®^ ACD tubes (Cat. no. BD 367986, Becton Dickinson, Franklin Lakes, NJ, USA) containing sodium citrate (22 g/L), citric acid (8 g/L), and dextrose (24.5 g/L). Then, 300 μM prostaglandin E1 (PGE1) diluted 1:10 (Cat. no. 13010.1, Cayman Chemical, Ann Arbor, MI, USA) was precisely added to each blood tube. The blood was centrifuged at 150× *g* for 15 min at room temperature, without interruption, to obtain platelet-rich plasma (PRP). The PRP was carefully transferred to 50 mL Falcon tubes, followed by the addition of an equal volume of sterile 0.4% EDTA solution (Cat. no. 114, Vetec Química Fina, Rio de Janeiro, Brazil) and PGE1 in a ratio of 1:1000. The samples were then centrifuged at 1000× *g* for 10 min at room temperature. The supernatant was discarded, and the platelet pellets were resuspended in 10 mL of sterile 0.4% EDTA containing PGE1 at a ratio of 1:1000. After gentle homogenization, the samples were centrifuged again at 1000× *g* for 10 min. The final pellet was resuspended in 10 mL of RPMI-1640 medium (Cat. no. 11875093, Gibco, Waltham, MA, USA) supplemented with 2.5% 1M HEPES (Cat. no. 15630080, Gibco) and 1% penicillin/streptomycin (Cat. no. 15140122, Gibco). Platelet counts were performed in a Neubauer chamber, using a 1:10 dilution of the cell suspension in filtered Rees-Ecker solution. Platelet counts were adjusted to 2 × 10^7^ platelets/mL in supplemented RPMI medium.

### 2.9. Platelet Stimulation and Activation

Initially, a volume equivalent to MOI (multiplicity of infection) 2 of the DENV-2 BR/RJ0337/2008 isolate was placed in contact with 40% inactivated plasma from pre- or post-COVID-19 dengue patients in a final volume of 200 μL of unsupplemented RPMI medium, followed by incubation for 1 h 30 min at 37 °C with 5% CO_2_. Then, 4 × 10^6^ platelets in 200 μL of supplemented RPMI medium were added in a volume of 200 μL under the conditions of DENV-2 pretreated with patient plasma. We used 4 × 10^6^ platelets in 400 μL supplemented RPMI medium as a negative control for platelet activation. As a positive control, we used 4 × 10^6^ platelets stimulated with 100 U/mL thrombin (Cat. no. T4393, Sigma-Aldrich, St. Louis, MO, USA) in a final volume of 400 μL of supplemented RPMI medium. To control viral infection, we used 4 × 10^6^ platelets initially stimulated with only DENV-2 isolate, without patient plasma, followed by adding 200 μL of supplemented RPMI medium. All conditions followed incubation for 30 min at 37 °C with 5% CO_2_. After incubation, samples were centrifuged at 1000× *g* for 10 min, and platelets were washed with 10 mL of supplemented RPMI 1640 medium and ready for extracellular labeling with monoclonal antibodies by flow cytometry.

### 2.10. Extracellular Labeling by Flow Cytometry

Previously stimulated platelets resuspended in 200 μL of blocking buffer and incubated for 30 min on ice. Then, platelets were centrifuged at 1000× *g* for 10 min at room temperature (RT), and a mixture of fluorochrome-conjugated monoclonal antibodies was added to the pellet: CD31-FITC (Cat. no. 9381-02, Biotech, Boston/Cambridge, MA, USA), CD41-PE-Cy5 (Cat. no. 303708, BioLegend, San Diego, CA, USA), and CD62P-APC (Cat. no. 304910, BioLegend), in addition to the cell viability dye LIVE/DEAD™ Fixable Green (Cat. no. L23101, Invitrogen; 1 μL) with incubation for 30 min at 4 °C, protected from light. Then, the samples were washed twice with wash buffer, centrifuging at 1000× *g* for 10 min at 4 °C. Finally, the platelet pellets, now perfectly prepared, were resuspended in 300 μL of PBS and immediately submitted to acquisition on a FACSymphony A5 flow cytometer from the Fiocruz Technological Platforms Network (PDTIS).

### 2.11. Statistical Analysis

Descriptive and correlational analyses, based on medians and proportions, were performed. The nonparametric Mann–Whitney test was used to compare the two groups. For comparisons between more than two groups, we utilized the One-Way ANOVA test associated with Kruskal–Wallis’s method. When Kruskal–Wallis’s test indicated significant differences, Dunn’s multiple comparisons test was applied. Demographic, laboratory, and clinical variables were analyzed standardized: the Kruskal–Wallis’s test was used for quantitative variables, such as age and days of symptom onset, and the chi-square test for categorical variables. *p* values > 0.05 were considered statistically significant. Our research was conducted using GraphPad Prism software version 10.6.1 (San Diego, CA, USA).

## 3. Results

### 3.1. Clinical and Laboratory Profile of Dengue Before and After COVID-19

Our comparative data between Dengue patients unexposed or exposed to COVID-19 unveil novel findings (Table 1). Post-COVID-19 patients were recruited later after the onset of the disease compared to those unexposed (*p* = 0.01). Among the patients’ symptoms, bleeding was more frequent in patients with Dengue before COVID-19 than in post-COVID-19 patients (*p* = 0.012). Compared to healthy controls, platelet counts were lower in Dengue, regardless of COVID-19 exposure. The frequency of patients with positive serology for IgM anti-DENV was higher (*p* = 0.005), while viral load detection by qRT-PCR was lower (*p* = 0.02) in patients with post-COVID-19 Dengue than in those who did not have COVID-19. Among the patients with detectable viral RNA by RT-qPCR, 71% were classified as infected with DENV-1 and 29% with DENV-4 in the 2016–2018 period. In 2023, DENV-2 predominated (80%), followed by DENV-1 (20%). An additional analysis was conducted to assess the relationship between viral serotype and platelet count, regardless of pandemic period. Patients infected with DENV-2 had lower mean platelet counts (133 × 10^3^/mm^3^) statistical difference compared to those with DENV-1 (212 × 10^3^/mm^3^), though no statistical difference was found with DENV-4 (156 × 10^3^/mm^3^). No significant differences were observed in the frequency of hemorrhagic manifestations among the different serotypes. Leukopenia (*p* = 0.001) and lymphocytopenia (*p* = 0.004) were preferentially observed in the post-COVID-19 dengue group compared to the unexposed ones. ALT levels were higher in the post-COVID-19 dengue group than in health controls (*p* = 0.031). Finally, AST levels (*p* = 0.001) and APRI ratio (*p* = 0.020) differentiated dengue groups, with those exposed to SARS-CoV-2 showing higher medians than those not exposed.

### 3.2. Change in Nitrite (NO_2_^−^) Levels in Dengue Before and After COVID-19

**Figure 1:**
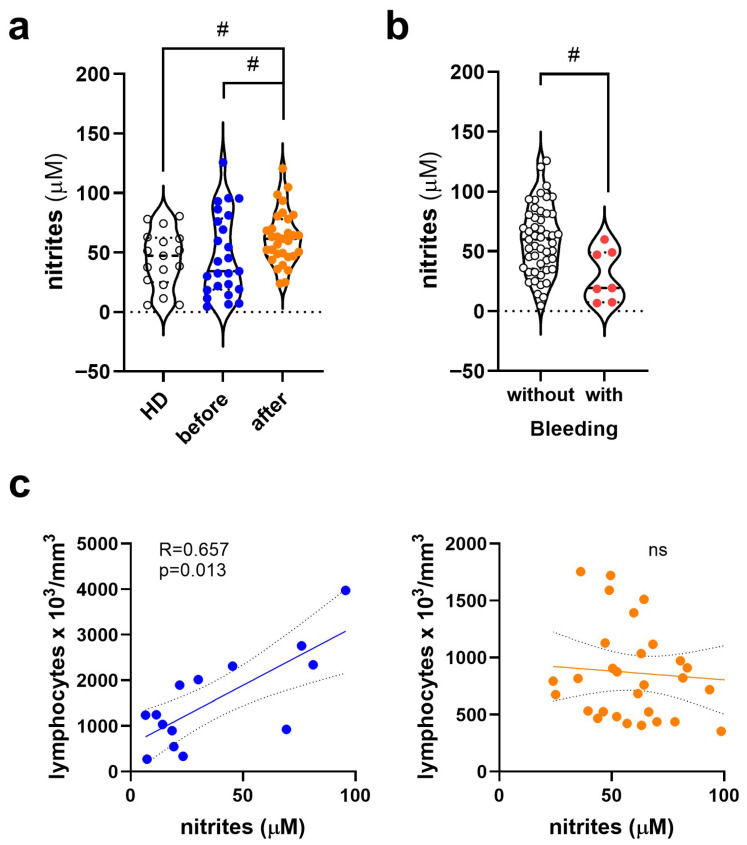
Plasma nitrite (NO_2_^−^) levels in healthy donors and dengue patients, with or without prior exposure to SARS-CoV-2. (**a**) Plasma nitrite levels in the three groups of participants: healthy donors (HD, white symbol, *n* = 17), dengue patients without prior exposure to SARS-CoV-2 (blue symbol, *n* = 32), and dengue patients exposed to SARS-CoV-2 (orange symbol, *n* = 36). (**b**) Plasma nitrite levels in patients without (white symbol, *n* = 49) and with hemorrhagic manifestations (red symbol, *n* = 7), regardless of exposure to SARS-CoV-2. Violin plots show the median (middle line) and describe numerical data distributions using density curves. (**c**) Correlation analysis between nitrite levels and lymphocyte counts in dengue patients before (blue symbol) and after COVID-19 (orange symbol). In (**a**), the nonparametric Mann–Whitney test was used to compare Dengue before and after COVID-19, the same used in (**b**). Correlation analyses used Spearman’s analysis, whose R > 0.500 and *p* < 0.05 values were considered strong correlations. The symbol “ns” means not significant. The symbol # represents values of *p* < 0.05, considered significant by the Mann–Whitney test.

Nitric oxide (NO) measurements by quantification of plasma nitrites (NO_2_^−^) between patients and healthy donors did not demonstrate a statistical difference compared to the Kruskal–Wallis’s test, followed by the Dunn test. However, analyses between the two groups of Dengue patients, by the Mann–Whitney test, indicated that, in Dengue, nitrite levels were increased in post-COVID-19 patients compared to patients not exposed to COVID-19 and healthy controls (Figure 1a). When categorizing patients according to bleeding, those who did not experience bleeding manifestations had increased nitrite levels compared to those who did (Figure 1b). Correlation analyses were performed between all quantitative and qualitative parameters, such as the detection or not of NS1 DENV antibodies, RT-qPCR DENV and IgM and IgG anti-DENV, as represented in Table 1, with nitrite levels. None of them were statistically significant. Nitrite levels directly correlated with lymphocyte counts in the Dengue group before COVID-19 (Figure 1c) but not in the Dengue group after COVID-19.

### 3.3. Changes in the Levels of Anticoagulant and Fibrinolytic Mediators in Dengue Before and After COVID-19

**Figure 2:**
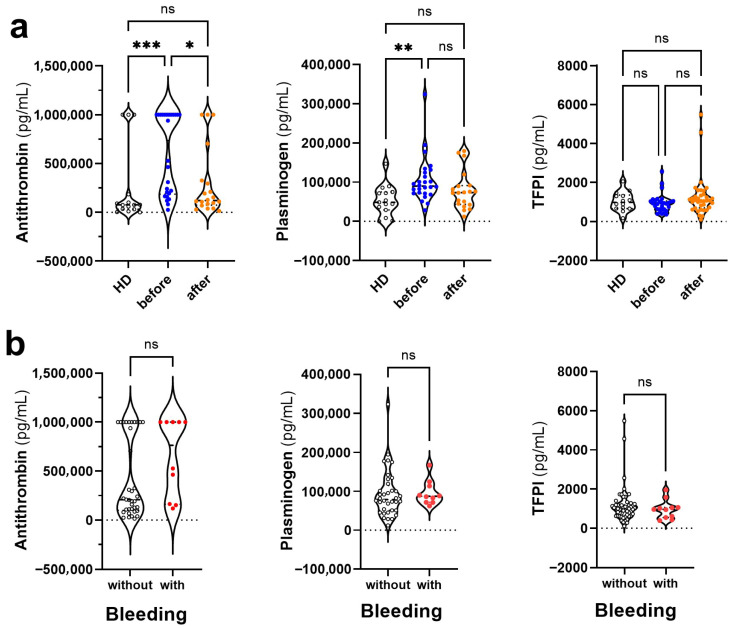
Levels of anticoagulant or fibrinolytic mediators in Dengue patients before and after COVID-19. (**a**) Plasma levels of antithrombin, plasminogen, and tissue factor inhibitor (TFPI) in the three groups of participants: healthy donors (HD, white symbol, *n* = 17), Dengue patients without prior exposure to SARS-CoV-2 (blue symbol, *n* = 32), and Dengue patients exposed to SARS-CoV-2 (orange symbol; *n* = 36). (**b**) These same mediators were evaluated in patients without (with symbol, *n* = 36) and with hemorrhagic manifestations (red symbol, *n* = 10), regardless of exposure to SARS-CoV-2. Violin plots show the median (middle line) and describe numerical data distributions using density curves. In (**a**), the nonparametric Kruskal–Wallis’s test was used, followed by Dunn’s post-test, and in (**b**), the nonparametric Mann–Whitney test. The symbol “ns” means not significant. The symbols *, **, and *** represent values of *p* < 0.05, <0.01, and <0.001, respectively, and were considered significant in all tests.

The observed thrombocytopenia in Dengue is independent of pre-exposure to SARS-CoV-2 (Table 1). Among the soluble mediators evaluated, we quantified proteins involved in anticoagulation and fibrinolysis, focusing on their potential modulation in dengue patients before and after COVID-19. As anticoagulant markers, we included antithrombin (AT) and tissue factor pathway inhibitor (TFPI). For fibrinolytic activity, plasminogen was the sole marker assessed. We found increased antithrombin levels in pre-COVID-19 Dengue compared to healthy donors and post-pandemic Dengue. Plasminogen was increased in the pre-COVID-19 Dengue group compared to healthy controls, while TFPI levels did not differentiate the groups (Figure 2a). The levels of the anticoagulant and fibrinolytic mediators did not differentiate patients with or without hemorrhagic manifestations (Figure 2b). Thus, in our analysis, we found that pre-COVID-19 dengue patients exhibited significantly higher levels of antithrombin and plasminogen than post-COVID-19 dengue patients, suggesting a potentially more anticoagulant and profibrinolytic profile in the earlier cohort. No significant differences were found in TFPI levels between the groups.

### 3.4. Changes in the Levels of Coagulation Mediators in Dengue Before and After COVID-19

**Figure 3:**
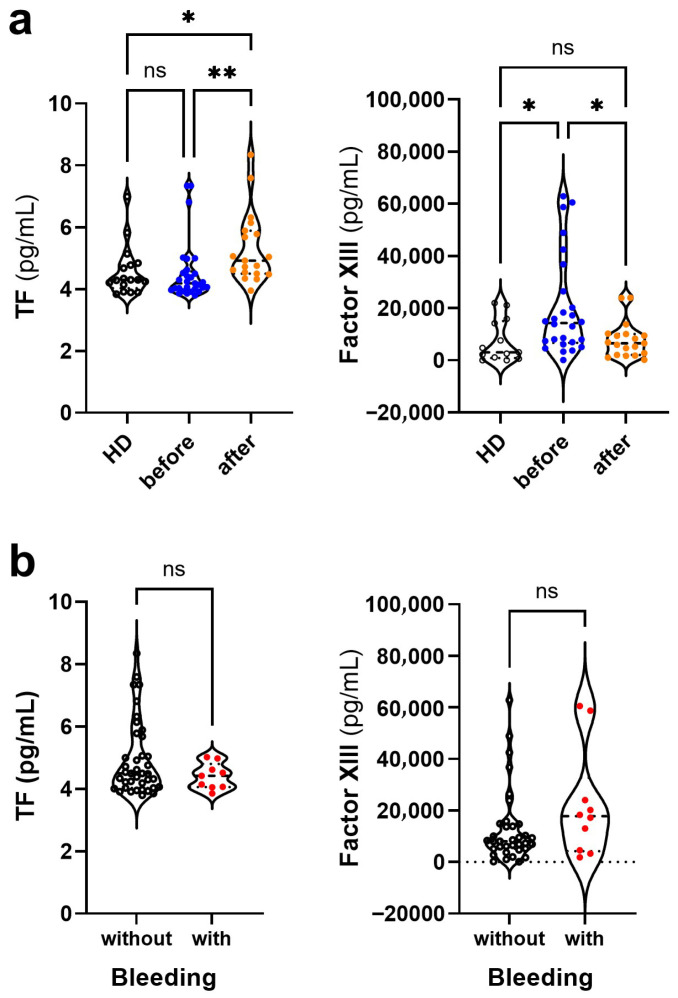
Levels of procoagulant mediators in dengue patients before and after COVID-19. (**a**) Plasma levels of tissue factor (TF) and factor XIII in the three groups of participants: healthy donors (HD, white symbol, *n* = 17), dengue patients without prior exposure to SARS-CoV-2 (blue symbol, *n* = 32), and dengue patients exposed to SARS-CoV-2 (orange symbol; *n* = 36). (**b**) These same mediators were evaluated in patients without (white symbol, *n* = 39) and with hemorrhagic manifestations (red symbol, *n* = 9), regardless of exposure to SARS-CoV-2. Violin plots show the median (middle line) and describe numerical data distributions using density curves. In (**a**), the nonparametric Kruskal–Wallis’s test was used, followed by Dunn’s post-test, and in (**b**), the nonparametric Mann–Whitney test. The symbol “ns” means not significant. The symbols * and ** represent values of *p* < 0.05 and <0.01, respectively, and were considered significant in all tests.

Unlike Dengue, COVID-19 is characterized by hypercoagulability [27]. Therefore, the coagulation mediators TF and Factor XIII levels were quantified in both groups of patients. Increased TF levels were preferentially observed in post-COVID-19 Dengue patients than in those before COVID-19 and healthy controls (Figure 3a). Conversely, increased levels of Factor XIII were observed in pre-COVID-19 Dengue than in post-pandemic Dengue and healthy controls (Figure 3a). These findings have significant implications for understanding the coagulation changes in Dengue patients’ post COVID-19. The levels of the two coagulation mediators did not differentiate between patients who did or did not have hemorrhagic manifestations (Figure 3b).

### 3.5. Platelet Activation Observed by CD62P Expression and NO Production

**Figure 4:**
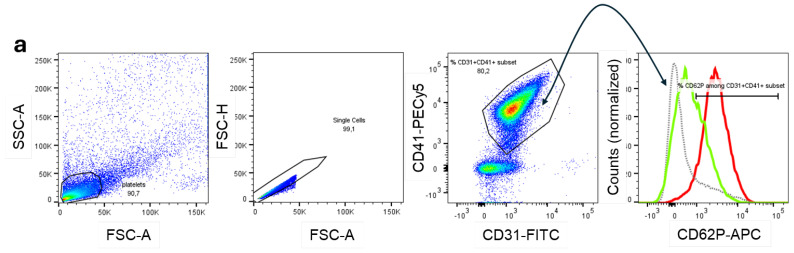

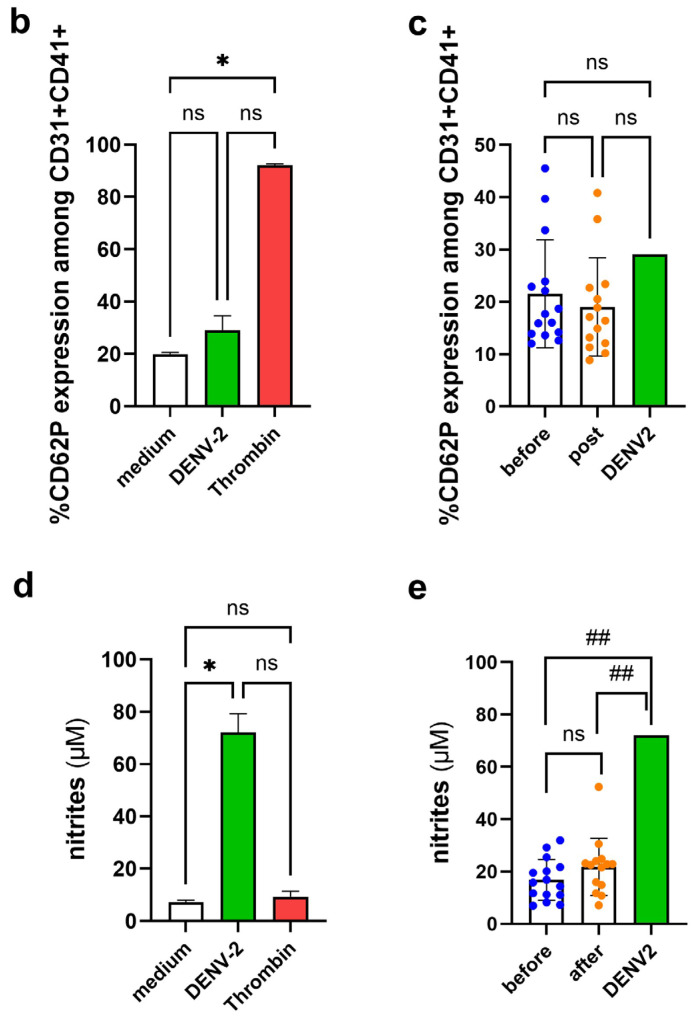
Platelet activation and nitrite production by platelets exposed to DENV-2 and plasma from dengue patients before and after COVID-19. (**a**) Representative analysis of the gating strategy was initially based on morphological analysis, followed by exclusion of doublets and selection of CD31^+^ and CD41^+^ populations. The histogram on the right shows the percentage of CD62P expression in CD31^+^ CD41^+^ platelets exposed to different conditions without the addition of patient plasma: culture medium (gray line), DENV-2 (green line), and thrombin (red line). (**b**) Analysis of platelet activation by CD62P staining in these three conditions without adding patient plasma. (**c**) CD62P expression was compared between platelets incubated with DENV-2 alone (gray bar, *n* = 3) or pretreated with plasma from dengue patients before (blue symbol, *n* = 15) and after COVID-19 (orange symbol, *n* = 14). (**d**) Nitrite production was quantified in the supernatant of platelets cultured in culture medium (medium; white), DENV-2 isolates (green), and thrombin (red). (**e**) Analysis of nitrite production in the DENV-2 isolate condition with plasma from patients before and after COVID-19. In (**b**–**e**), the nonparametric Friedmann’s test was used, followed by Dunn’s post-test. The symbol “ns” means not significant. The symbols # and * indicate values of *p* < 0.05, considered significant by the Mann-Whitney and Kruskal-Wallis tests, respectively.

After a process of separating platelets from healthy donors (*n* = 3), they were cultured in a culture medium, thrombin, DENV-2 isolate (MOI 2), and plasma from patients previously incubated with the DENV-2 isolates. After incubation, platelets were recovered, and the platelet activation marker CD62P expression on platelets and the NO level in the culture supernatant was assessed by flow cytometry and Griess assay, respectively. The morphological region of platelets, followed by singlet selection, was initially analyzed, and the percentage of donor platelets constituted more than 90% of all cells in the cell suspension. Singlets were delimited and separated from smaller particles based on size and complexity. The population representing >90% of singlets was examined for co-expression of CD31 and CD41 to ensure that the resulting population corresponded to platelets. Subsequently, within the population expressing CD31 and CD41, the CD62P molecule was evaluated as an indicator of surface platelet activation. The histogram shows a single population stained with CD62P under medium, DENV-2, and thrombin conditions (Figure 4a). After treatment of platelets with DENV-2 alone, a small increase percentage of platelets expressing CD62P was found compared with platelets grown in culture medium. Thrombin treatment increased CD62P expression more than the other treatments (Figure 4b). Under conditions where DENV-2 was previously incubated with plasma from patients before and after COVID-19 and then added to platelets, CD62P expression was similar between the two groups of dengue-patients and not differentiate from DENV-2 alone (Figure 4c).

NO is another marker that influences platelet functionality [28]. In platelet infection with DENV-2 alone, a marked increase in NO was observed compared to culture medium and thrombin conditions (Figure 4d). Plasmas from before and after COVID-19 patients pre-incubated with DENV-2 and subsequently added to platelets showed no difference in NO production in platelet cultures between the two groups. However, the addition of plasma significantly decreased NO production compared to platelets cultured with DENV-2 alone (Figure 4e).

## 4. Discussion

Previous studies have shown that SARS-CoV-2 infection can cause false-positive results in rapid dengue serological tests [11,12], but the impact of pre-existing dengue immunity on COVID-19 severity remains unclear. Post-COVID-19 antibodies against SARS-CoV-2 may either protect against dengue infection by cross-reacting with DENV or potentially enhance it through antibody-dependent enhancement (ADE) [16]. Supporting this, Chen et al. found increased anti-SARS-CoV-2 antibodies in dengue patients, which showed neutralizing effects against DENV in vitro, suggesting a milder dengue presentation. Conversely, a study from northern India reported that prior COVID-19 infection increases the risk of symptomatic and severe dengue, despite no effect of COVID-19 vaccination on dengue severity [29]. Inspired by Chen et al.’s finding that passive transfer of anti-S1-RBD IgG reduced bleeding time in dengue-infected mice [9] and given the common occurrence of thrombocytopenia and platelet dysfunction in both dengue and COVID-19 [30], we analyzed an original dataset comparing acute dengue patients with and without prior COVID-19 exposure.

Our data show similar thrombocytopenia levels in dengue patients before and after COVID-19, but hemorrhagic symptoms were less frequent post COVID-19. This suggests that prior COVID-19 exposure may improve hemostasis in dengue. Understanding these changes is crucial, especially since anticoagulants used in COVID-19 treatment [31] are contraindicated in dengue.

Considering the fundamental role that nitric oxide plays in a wide variety of processes, such as a neurotransmitter, vasodilator, and platelet aggregator, in the fight against infections and tumors, as an inflammatory and cytotoxic mediator of macrophages, among others [32,33], we evaluated the indirect measurement of its production by determining the levels of its metabolites, nitrites.

Oxidative stress is linked to COVID-19 thrombotic complications [34]. In dengue, platelets produce higher nitric oxide (NO) levels during the acute phase, associated with warning signs and hemorrhagic symptoms, driven by IL-1 receptor signaling [35]. Conversely, COVID-19 patients with thrombosis show reduced platelet NO production [36]. Although we observed increased nitrite levels in post-COVID-19 dengue patients, particularly in those without hemorrhagic manifestations, we interpret this finding with caution. The dual role of NO in dengue pathophysiology is well-documented; it may exert beneficial effects by promoting vasodilation and inhibiting platelet activation [37], yet excessive NO production can also enhance vascular permeability and contribute to tissue damage [38]. Therefore, the observed association between NO and clinical manifestations in our cohort should be viewed as hypothesis-generating and warrants further mechanistic investigation.

The hemostatic phenotype of infectious diseases, i.e., blood coagulation and fibrinolysis, is variable and essentially activated based on the strategy of different infectious agents. COVID-19 is among contagious diseases with a prothrombotic phenotype, while dengue is characterized by a profibrinolytic behavior [39].

Dengue virus infects monocytes, triggering cytokine release and tissue factor (TF) expression, which activates coagulation. Endothelial damage also causes release of tissue plasminogen activator (t-PA), promoting fibrinolysis. Dengue patients with hemorrhagic complications show thrombocytopenia and reduced thrombin generation, reflecting a balance tilted toward hyperfibrinolysis despite coagulation activation [40]. Our study found increased antithrombin and plasminogen in pre-COVID-19 dengue patients, but not TFPI, compared to post-COVID-19 patients and controls. Previous research identified antithrombin antibodies in dengue, mostly IgG targeting DENV NS1 and plasminogen [41].

An important limitation of this study relates to the fact that data were obtained from two distinct time periods (2016–2018 vs. 2023), which may introduce temporal and epidemiological bias, particularly due to variations in circulating DENV serotypes, the proportion of primary vs. secondary infections, and epidemic dynamics. Indeed, we observed differences in serotype distribution across periods: DENV-1 and DENV-4 predominated before the pandemic, whereas DENV-2 was the major circulating serotype in 2023. Previous studies by our group demonstrated that DENV-2 infections were associated with lower platelet counts and more severe clinical manifestations, including hemorrhagic symptoms, when compared to DENV-4 [42]. However, despite the higher prevalence of DENV-2 in the post-COVID-19 period (2023), we did not observe more severe clinical outcomes or significantly lower platelet counts compared to the pre-pandemic period. These findings suggest that while seasonal and serotype variations can influence dengue clinical outcomes, other immunological and contextual factors—such as prior exposure to SARS-CoV-2—may also play an important role in modulating inflammatory and hemostatic responses.

Most post-COVID-19 dengue patients reported both previous SARS-CoV-2 infection and vaccination. Exposure was confirmed by detection of IgG against the Spike S1 protein, which does not differentiate natural infection from vaccination—a limitation of our study. Considering the epidemiological context in Brazil during 2021–2023, characterized by high vaccination coverage and widespread viral circulation, separating these subgroups was not feasible in our cohort. Sample size limitations were acknowledged and taken into consideration when interpreting the findings.

We acknowledge that the later recruitment of patients in the post-COVID-19 group, likely influenced by healthcare-seeking behavior during the pandemic, may have affected biomarker levels due to temporal changes in inflammatory and vascular responses. Additionally, the potential impact of medications used for COVID-19 or comorbidities (e.g., hypertension, diabetes, corticosteroids, anticoagulants) on hemostatic markers cannot be excluded. However, detailed therapeutic data were not uniformly available, representing a limitation of the study. Future research will aim to incorporate more comprehensive medication profiling to allow for adjusted analyses.

SARS-CoV-2 uses its Spike S1 protein to bind ACE2 receptors [43], causing cytopathic effects and triggering cytokine release and monocyte activation, like dengue, which leads to tissue factor (TF) expression and blood clotting [44]. Similarly, the differences in TF and Factor XIII levels between pre- and post-COVID-19 dengue patients are intriguing but should not be overinterpreted as definitive evidence of post-COVID-19 sequelae [45]. While previous studies have shown that SARS-CoV-2 can induce persistent endothelial dysfunction and alter coagulation pathways [46,47], we acknowledge that other factors—such as dengue serotype variation, host immune status, and timing of sample collection—may also contribute to these findings. Thus, we now emphasize the preliminary nature of these observations and the need for longitudinal or functional studies to explore whether these hemostatic changes reflect long-term immune or endothelial alterations following SARS-CoV-2 exposure.

## 5. Conclusions

From a translational perspective, the modulation of hemostatic markers such as Tissue Factor (TF), Factor XIII, and nitric oxide (NO)-derived nitrites in dengue patients’ post COVID-19 may offer insights for clinical monitoring strategies. TF is a key initiator of the extrinsic coagulation pathway, and its increased levels could reflect a more procoagulant state, potentially informing the risk of thrombosis in certain dengue cases. Conversely, reduced levels of Factor XIII—responsible for stabilizing fibrin clots—have been associated with more severe bleeding in viral infections, suggesting that its monitoring may assist in predicting hemorrhagic complications. Nitrites, as a surrogate for NO, could also serve as markers of endothelial activation or dysfunction, with potential to discriminate between patients with different inflammatory or vascular responses. While these biomarkers are not currently used in routine dengue care, our findings suggest they may contribute to future risk stratification models in the post-COVID-19 era, especially for patients with complex immunological backgrounds due to prior SARS-CoV-2 exposure.

## Figures and Tables

**Table 1:** Demographic, clinical and laboratory characteristics of healthy donors exposed to COVID-19 and patients with Dengue before and after COVID-19.

	HD Post-COVID-19 (*n* = 17)	Dengue Before COVID-19 (*n* = 32)	Dengue Post-COVID-19 (*n* = 36)	*p* Value
Gender (female: male) ^a^	14:3	17:15	23:13	ns
Age, years ^b^	31 (24–60)	39 (21–88)	38.5 (18–86)	ns
**days after the onset of symptoms ^c^**	**NA**	**3 (1–8)**	**5 (2–7)**	**0.01**
**Signs and symptoms (%) ^d^**				
fever	*NA*	26 (81)	26 (72)	ns
headache	*NA*	27 (84)	22 (61)	ns
myalgia/arthralgia/low back pain	*NA*	30 (94)	28 (78)	ns
retroorbital pain	*NA*	19 (59)	17 (47)	ns
vomiting/nausea/abdominal pain	*NA*	21 (66)	18 (50)	ns
Fluid accumulation	*NA*	0	0	ns
**Bleeding**	**NA**	**9 (28)**	**2 (6)**	**0.012**
Dengue Fever	*NA*	23 (72)	26 (72)	ns
Dengue with Warning Signs	*NA*	9 (28)	10 (27)	
**Serological and molecular diagnosis (%) ^d^**				
IgG anti-DENV	13 (76)	23 (70)	31 (86)	ns
IgM anti-DENV	0	17 (53)	19 (54)	ns
NS1 DENV Ag	0	21 (66)	29 (81)	ns
RT-qPCR DENV	*NA*	17 (53)	7 (19)	**0.02**
**Blood Count (×10^3^/mm^3^) ^b^**				
Platelet	292 (200–367)	199 (59–356)	170 (55–358)	HD vs. Dengue not-exposed *p* = 0.003 HD vs. Dengue exposed *p* < 0.001
Leucocyte	6600 (4680–9690)	5000 (2100–19,700)	2850 (1500–5100)	HD vs. Dengue exposed *p* < 0.001 **Dengue not-exposed vs. exposed *p* < 0.001**
Lymphocyte	2150 (1002–32,000)	1452 (272–3975)	816 (354–1754)	HD vs. Dengue not-exposed *p* < 0.001 **Dengue not-exposed vs. exposed *p =* 0.004**
Monocyte	390 (243–3000)	366 (22–985)	355 (71–612)	ns
ALT (IU/L) ^b^	17 (6–54)	27 (10–76)	33 (13–1152)	HD vs. Dengue exposed *p* = 0.031
AST (IU/L) ^b^	21 (13–52)	26 (11–111)	51 (21–438)	HD vs. Dengue exposed *p* = 0.006 **Dengue not-exposed vs. exposed *p =* 0.001**
APRI ^b^	0.06 (0.04–0.20)	0.10 (0.03–1.88)	0.32 (0.07–6.60)	HD vs. Dengue exposed *p* = 0.020

The total study population consisted of 85 participants, distributed among healthy donors exposed to COVID-19 by natural infection and/or vaccination (HD, *n* = 17), dengue patients never exposed to SARS-CoV-2 recruited in 2018 (*n* = 32), and dengue patients exposed to SARS-CoV-2 by natural infection and/or vaccination recruited in 2023 (*n* = 36). (a) Gender was expressed as the proportion of females and males with distribution analysis using the nonparametric chi-square exact test. (b) Age, blood count, transaminases, and APRI were presented as medians with minimum-maximum intervals using the Kruskal–Wallis’s test, followed by Dunn’s multiple comparisons test. (c) Days after disease onset were presented as median, with minimum-maximum intervals, using the Mann–Whitney test. (d) Signs, symptoms, and molecular and serological diagnoses were presented as the number of positive cases and the percentage in parentheses. The statistical test used was Fisher’s exact test. Bleeding manifestations refer to mild mucocutaneous events (e.g., petechiae, epistaxis, gingival bleeding). No gastrointestinal or central nervous system bleeding was recorded. Fisher’s exact test was used for these variables. The term “NA” was used to indicate non-applicable variables and “ns” when the statistical test was considered non-significant or with a *p*-value > 0.05.

## Data Availability

The data presented in this study is available on request from the corresponding author.
